# Unilateral pedal lymphangiography with non-contrast computerized tomography is valuable in the location and treatment decision of idiopathic chylothorax

**DOI:** 10.1186/1749-8090-9-8

**Published:** 2014-01-07

**Authors:** Ding-Yi Liu, Yuan Shao, Jian-Xin Shi

**Affiliations:** 1 Department of Urology, Shanghai Pu-nan Hospital, 2400, South Pudong Road, Shanghai 200125, China; 2 Department of Urology, Ruijin Hospital, School of Medicine, Shanghai Jiaotong University, 197 Ruijin Er Road, Shanghai 200025, China; 3 Department of Thoracic Surgery, Shanghai Chest Hospital Affiliated to Shanghai Jiaotong University, 241 West Huaihai Road, Shanghai 200040, China

**Keywords:** Idiopathic chylothorax, Lymphangiography, Unilateral, Thoracic duct

## Abstract

**Purpose:**

To identify the value of unilateral pedal lymphangiography (LAG) with non-contrast CT in the location and treatment decision of idiopathic chylothorax after failure of thoracic duct ligation.

**Materials and Methods:**

Twenty four patients aged 9–84 year old (median 44 yr) who had idiopathic chylothorax were involved, and unilateral pedal LAG with non-contrast CT was performed in every patient. All patients failed to previous right supra-diaphragmatic thoracic duct ligation.

**Results:**

The amount of iodized oil used was 6–14 ml with no related complications. LAG demonstrated 8 patients with thoracic duct leaks and 10 patients with leaks elsewhere, but no visible chylous leak in 6 patients. Ligation of thoracic duct was performed as the primary treatment in all 8 cases as having thoracic duct leakage and cured 7(87.5%) patients. For 8 patients not having thoracic duct lesion under LAG, the successful rate of thoracic duct ligation was 25% (2 out of 8 patients), which was significantly lower than patients due to thoracic duct lesions (*P* = 0.02). Meanwhile, non-operative therapy had significantly higher successful rate (87.5% vs 25%, *P* = 0.02).

**Conclusions:**

Unilateral pedal LAG with non-contrast CT could identify the causes and locate the leaks of idiopathic chylothorax in 75% of patients after failure of thoracic duct ligation. Two thirds of patients were found not to have thoracic duct leakage and would be better managed by non-operative treatment.

## Background

Chylous pleural effusion, or chylothorax, is defined as the accumulation of chyle-containing lymphatic fluid within the pleural space [1]. It has various causes and is usually attributable to 1 of the 4 categories: malignancies, trauma (including surgery), miscellaneous disorders and idiopathic [2]. Idiopathic chylothorax is rare, which may occupy 6.4% of all chylothorax patients [1], and includes most cases of congenital variety as well as other cases with no clear cause [2].

Despite of its rarity, extensive chyle leakage from idiopathic chylothorax can also have deleterious physiologic consequences in the form of hypovolemia and hypoproteinemia resulting from severe fluid and protein loss [3]. Historically, fluid sampling, surgical exploration, and various imaging modalities, including lymphangiography (LAG), computed tomography (CT), magnetic resonance imaging (MRI), and nuclear imaging have all contributed to the diagnosis and localization of chylothorax. However, LAG combined with post-LAG CT imaging is superior in depicting and precisely localizing chylous abnormalities, while LAG has also been shown to be therapeutic in some instances [4-8]. Although bipedal LAG is recommended to demonstrate the anatomy of the thoracic duct and to indicate the cause of chylothorax [9], the diagnostic capabilities of unilateral pedal or monopedal LAG has also been demonstrated in the literature [10].

If conservative treatment of chylothorax is unsuccessful, or in the event of high flow chylothorax surgical ligature of the thoracic duct must be undertaken [11]. The purpose of our study was to analyze the results of unilateral LAG with post-LAG non-contrast CT scan in idiopathic chylothorax patients after failure of right supra-diaphragmatic thoracic duct ligation, aiming to identify its value in proper location and treatment guidance of these refractory cases.

## Methods

From year 2000 to 2011, twenty-four patients aged 9–84 year old (median 44 yr) who had idiopathic chylothorax were involved in this series, including 10 males and 14 females. All patients failed to their previous right supra-diaphragmatic thoracic duct ligations. Main clinical manifestations were dyspnea, shortness of breath or unavailable of prostration. The presence of chylothorax was defined by 1 or more of the following inclusion criteria: (1) a pleural fluid triglyceride level of 110 mg/dL or greater; (2) the presence of chylomicrons in the pleural effusion. Other causes of chylothrax, such as malignancies, trauma, tuberculosis or filariasis, were excluded. After their previous thoracic duct ligations, 20 patients had unilateral (9 in left and 11 in right side) and 4 patients had bilateral chylothorax with duration of 3 days to one year. Conventional unenhanced MRI was done for each patient but only located suspected leak sites of thoracic duct in 4 patients. Thus, patients were screened for contraindications to LAG, including cardiovascular and pulmonary disease (i.e., right-to-left shunts, pulmonary insufficiency, etc.) [4]. Risks, benefits, and alternative procedures were discussed with each patient or custodian, and written informed consent was obtained in accordance with institutional ethical policy before performing LAG. This study was approved by the ethics committee of all authors' hospitals, and informed consent was obtained from all patients at the time of enrolment.

Unilateral pedal LAG was performed according to the following steps. First, 2 ml of methylene blue, a dye that stains the lymphatics, was injected into the web space between the first and second toes of one foot. Thirty minutes later, linear cut-down was performed on the dorsum of the foot below the ankle, and the lymphatic vessel was isolated. After cannulation of a lymphatic vessel on the dorsum of foot using a 30 gauge needle, iodized oil (Lipiodol; Laboratoire Guerbet, Roissy, France), which is a contrast agent for LAG, was injected at a rate of 0.1 ml/min, not exceeding a total volume of 14 ml.

Completion radiographs were obtained to assess for immediately apparent chyle leaks and to document the filling phase of the LAG. Each patient had follow-up nodal phase radiographs 24 h after the initial procedure. In these nodal phase radiographs, the pleural space, peritoneal space, and urinary collecting system were examined for signs of contrast extravasation. Any readily apparent leaks were reassessed and any newly apparent leaks were documented. In all cases, a non-contrast CT scan was also performed at that time to confirm the result of LAG or determine the exact level of the lymphatic leakage.

## Results

**Figure 1:**
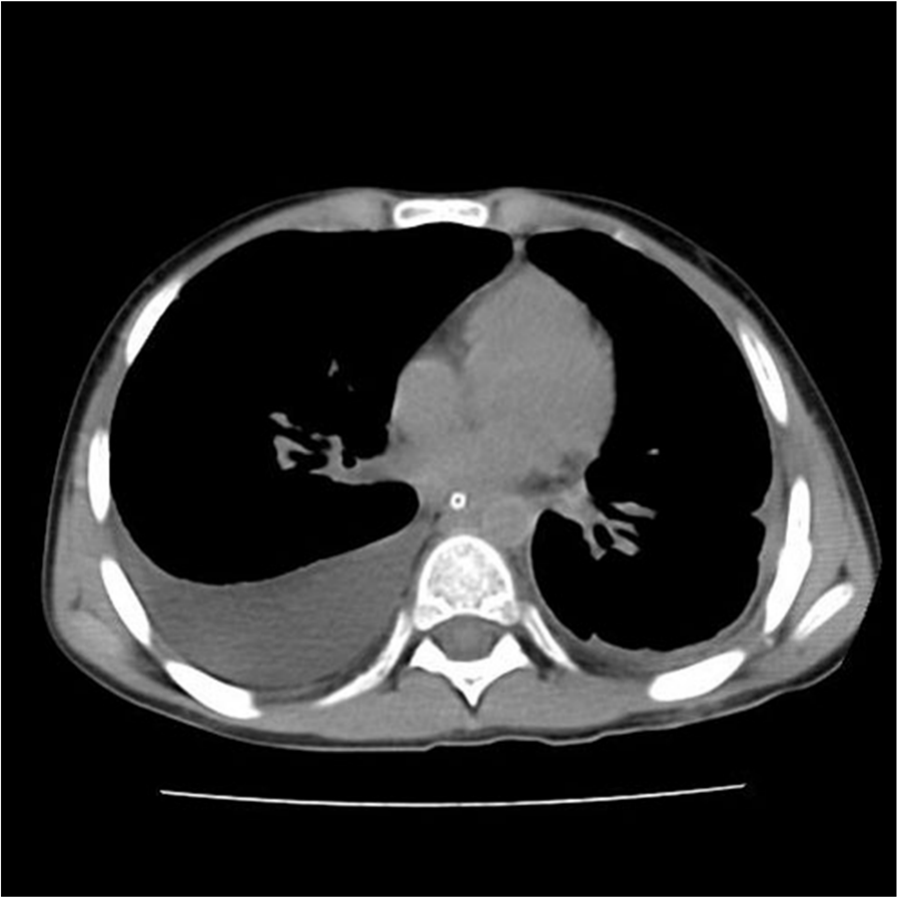
Pre-LAG CT of a nine-year-old boy had bilateral chylothorax after thoracic duct ligation.

**Figure 2:**
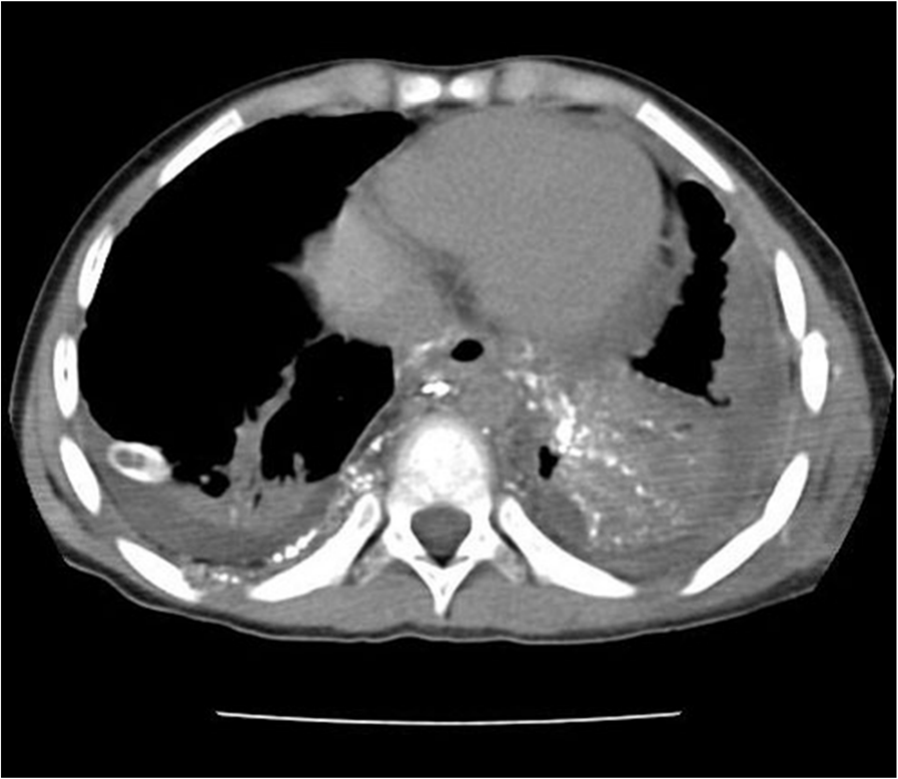
Extensive thoracic lymphatic leakage detected by post LAG non-contrast CT.

**Figure 3:**
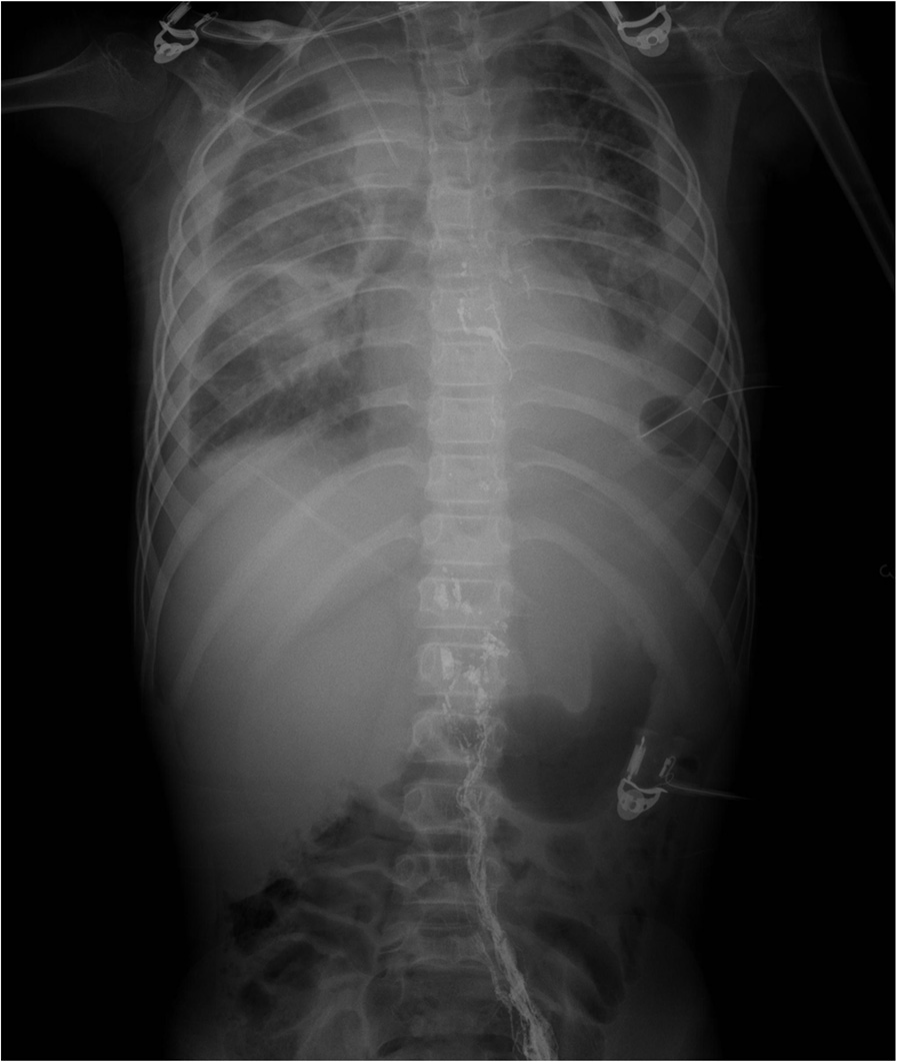
LAG of this nine-year-old boy showed no leakage of thoracic duct.

All 24 patients underwent unilateral pedal LAG with the amount of iodized oil from 6 to 14 ml. LAG was successful in all patients with no related complications. Of these patients, LAG showed chylous leakage from thoracic duct in 8 (including those 4 patients whose chylothorax was detected by MRI) and from intra- or retroperitoneal lymphatics in 7 and 2 patients respectively, whereas 6 patients showed no chylous leak by LAG with CT. The remaining patient, a 9-year-old boy who had bilateral chylothorax was detected to have extensive pleural lymphatic leak (Figures 1, 2 and 3). The duration of follow-up varied from 3 to 108 months (median 12 months) after LAG.

LAG with non-contrast CT properly located chyle leak in 18 (75%) of all 24 cases, whereas 6 patients showed no leakage. (Table 1) For patients due to thoracic duct leakage, repeated ligations of thoracic duct were performed in all 8 cases with the successful rate of 87.5% (7 out of 8 patients). For those patients with their origin other than thoracic duct lesions under LAG, thoracic duct ligation was performed in 8 but only cured or improved 2 (25%) patients. Its successful rate was significantly lower than in patients with thoracic duct leakage (25% vs 87.5%, *P* = 0.02). The remaining 8 patients not due to thoracic duct leaks received non-operative therapy with thoracentesis. Its cure or improve rate was 87.5% (7 out of 8 patients), which was significantly higher than thoracic duct ligation (87.5% vs 25%, *P* = 0.02) (Table 1).

**Table 1:** Therapeutic methods and outcome in patients with idiopathic chylothorax

**Chylothorax classified by LAG + CT**	**Treatment performed**	**Outcome**
Thoracic duct leakage (8)	Second thoracic duct ligation (8)	Cured (7)
		Failed (1)
Extensive pleural lymphatic leak (1)	Second thoracic duct ligation (1)	Failed (1)
Intra/retroperitoneal lymphatic lesions (9)	Thoracentesis + conservative (5)	Cured (2), improved (2) failed (1)
	Second thoracic duct ligation (4)	Cured (1) failed (3)
Normal LAG (6)	Thoracentesis + conservative (3)	Cured (2), improved (1)
	Second thoracic duct ligation (3)	Improved (1) failed (2)

Data presented as number of patients in parentheses.

## Discussion

Idiopathic chylothorax is a rare condition with unclear cause. It can develop as a result of ruptured of dilated thoracic duct, with or without proximal occlusion, or as a result of rupture of diaphragmatic or intercostal lymphatic collaterals, or as a result of pulmonary lymphangiomyomatosis [12]. Patients who failed to thoracic duct ligation tended to be obscure and difficult cases, requiring accurate identification and location of the chylous leakage.

Lymphoscintigraphy is performed by injecting technitium-99 m labeled Sb2-S3 colloid or labeled human albumin into the interdigital space of the foot or hand. By employing semiquantitative transport index, a 92% sensitivity and nearly 100% specificity can be achieved in diagnosing lymphedema [13,14]. Since lymphoscintigraphy is a functional study, we performed LAG with non-contrast CT to define lymphatic anatomy and the site of lymphatic leak.

Recently, the feasibility of MR lymphography has been established by performing multistation imaging of the lower extremities with three-dimensional spoiled gradient echo sequences after a cutaneous injection of gadodiamide. It has proved to be a safe, noninvasive, high-resolution technique for depicting lymphatic abnormalities without using ionizing radiation [15]. Evaluation of abdominal and retroperitoneal lymphatic abnormalities, including lymphatic leaks, has also been described through MR lymphography through heavily T2-weighted fast spin-echo sequences [16]. Despite these advances, conventional LAG remains the gold standard in the evaluation of chyle leaks due to its ability to opacify the lymphatic channels and highlight the presence of lymphatic fistulae or leakage [4].

Although bipedal LAG is recommended to demonstrate the anatomy of the thoracic duct and to indicate the cause of the chylothorax [9], the diagnostic capabilities of unilateral pedal LAG has also been demonstrated. Fistulas can often be identified by way of crossover channels that divert lymphatic flow from the contralateral lymphatics toward the fistula [10]. Koga et al. reported lymphatic vessels crossing from the injected to the uninjected side occurred in all 106 patients with chyluria. The direction of flow in the lymphatic channels, being a closed circulation system with a positive pressure, will converge to the point where the integrity of the channels has been breached. So unilateral LAG can detect chyle leakage even when it is on the side opposite that where contrast is injected [10]. The advantages of unilateral over bilateral LAG are that it is easy to identify crossover channels, and there is less discomfort for the patient because of fewer incisions and quicker procedure [10].

In our study, all 24 patients received monopedal LAG with their thoracic or abdominal lymphatic vessels being shown well. LAG detected thoracic duct leaks in 33.3%, extensive pleural leakage in 4.2%, intra- or retroperitoneal lymphatic leaks in 37.5% and no abnormality in 25% of patients as the origins. This result indicated that the majority of idiopathic chylothorax with thoracic duct ligation failure could be intra- or retroperitoneal leaks, which was different to previous reports [12].

The number of conventional LAGs has declined markedly since the introduction of cross-sectional imaging techniques. Nevertheless, LAG has a high potential as a reliable method to visualize and directly occlude lymphatic leaks. In nearly 79% of patients, the location of the leak could be detected, and surgical intervention could be planned when therapeutic LAG failed [17]. Due to the irrigating effect of Lipiodol, the lymphatic leak could be completely occluded in 70% of patients when the lymphatic drainage volume was less than 500 mL/day. Even when lymphatic drainage was higher than 500 mL/day, therapeutic LAG was still successful in 35% of the patients. The overall success rate in patients with failed conservative treatment was 51% [17]. The speculated mechanism of attenuation of chyle leakage was thought to be as follows. Firstly, Lipiodol infused during lymphangiography accumulated at the point of leakage outside the lymphatic vessel. Secondly, a regional inflammatory reaction occurred in the soft tissue adjacent to the area of Lipiodol retention. Thirdly, the point of leakage of the lymphatic vessel was obstructed. Finally, Lipiodol retention inside the lymphatic vessel on the distal side of the point of leakage played a role as a therapeutic embolic agent [5,6].

In our series, LAG with non-contrast CT detected and located chyle leaks in 75% of patients. Its finding could provide important information in decision of treatment. In cases with underlying causes other than thoracic duct lesions by LAG with CT, non-operative management with thoracentesis had a better cure or improve rate than thoracic duct ligation (87.5% vs 25%, P = 0.02). We suggest that unilateral pedal LAG with non-contrast CT is valuable to determine underlying lymphatic vessel leak and localize the leakage site for surgical therapy. It could also indicate those patients suitable for non-operaitve treatment to avoid unnecessary surgical interventions.

Infection and pain are the most common complications seen with LAG, but serious complications have also been reported, including intra-alveolar hemorrhage, allergic reactions to Lipiodol or methylene blue, extravasation of contrast into the deep tissues of the foot, and oil emboli to the lungs, brain, kidney, and liver [3,5,8,18-20]. Fortunately, there was no detectable complication in our series.

The limitation of this study is the limit number of patients with no randomized control comparison, predominantly due to the rarity of idiopathic chylothorax. A multicenter, randomized control study with large number of patients is necessary to investigate the exact value of unilateral pedal LAG with CT in the location and management of idiopathic chylothorax.

## Conclusions

Unilateral pedal LAG with non-contrast CT could identify the causes and leakage sites in 75% of patients with idiopathic chylothorax after failure of thoracic duct ligation. Two thirds of patients were found not to have thoracic duct leakage and would be better managed by non-operative treatment.

## Competing interests

The authors declare that they have no competing interests.

## Authors’ contributions

DL participated in the design of the study, LAG performing and helped to draft the manuscript. YS participated in the design of the study, the statistical analysis and draft the manuscript. JS performed thoracentesis and thoracic surgeries. All authors read and approved the final manuscript.
